# Exploring the Evidence for the Paradigms of Recovery and Social Work Converging in Mental Health Service Delivery Worldwide: Reflections from an Irish Case Study

**DOI:** 10.3390/ijerph20156460

**Published:** 2023-07-27

**Authors:** Calvin Swords, Stan Houston

**Affiliations:** 1 Department of Applied Social Studies, National University of Ireland, W23F2H6 Maynooth, Ireland; 2 School of Social Sciences, Education and Social Work, Queen’s University Belfast, Belfast BT71HL, UK; s.houston@qub.ac.uk

**Keywords:** recovery, mental health services, social work, critical social work, neoliberalism, social constructionism, case study

## Abstract

Recovery within mental health service delivery is no longer a new consideration in the Western world. However, it is well-documented how challenging its implementation and translation to practice and reality have been in contemporary mental health systems. In conjunction with this, mental health social work is continuously being challenged and debated in relation to its role, responsibilities, and identity in service delivery. This is largely the consequence of the continued dominance of the biomedical model in relation to service delivery. Yet, if we critically reflect on the philosophy and ethos of recovery, it becomes very clear that social work should be the key profession to lead the development and improvement of recovery-orientated services across the globe. To illustrate this argument, the authors first draw on empirical research undertaken by the lead author within the Republic of Ireland on how recovery is socially constructed within mental health service delivery. The key stakeholders involved in the Irish study included professionals, service users, family members, and policy influencers, with participants taking part in semi-structured interviews. Secondly, the authors reflect on some of the findings from this Irish study, presenting an argument for not only a more significant role for social work in an Irish mental health context but also making comparisons from an international perspective. This includes exploring the role of critical social work traditions for supporting services to move beyond a philosophy of recovery that has, to date, overlooked the intersectional injustices and inequalities faced by hard-to-reach populations. Finally, the authors conclude by providing some possibilities for how the paradigms of social work and recovery can and should continue to converge towards each other, opening a space for social work to become a more dominant perspective within mental health systems worldwide.

## 1. Introduction

In 2023, the concept of recovery as a personalised journey is well-documented and discussed across mental health services [1,2,3,4]. Recovery as a personalised journey is focused on supporting people with diagnoses to live satisfying, contributing, and meaningful lives beyond the label of mental illness [5]. It is a unique, subjective experience, which can only truly be measured by each individual living with a mental illness [6,7,8].

Until the late 20th century, psychiatry and prescribed medication were the dominant discursive practices in mental health service delivery [9,10]. Up until this point, recovery was constructed within service culture as a physical illness, which was treated through medication [1,2,3,4]. In the 1980s and 1990s, the rise of the survivor movement and personal narratives of recovery contributed to new interpretations of recovery [2,11]. More specifically, individuals using services were beginning to challenge the model of service delivery through their personal accounts and collective movements, claiming that recovery is possible beyond the medical model [1,2,12].

This included the construction of recovery as a personalised journey through life, regardless of the label of mental illness [4]. In 1993, a commonly used definition of personal recovery was published [12]:

*“It is way of living a satisfying, hopeful and contributing life, even with limitations caused by illness.”*

*([12], p. 15)*

This new way of viewing recovery in mental health service culture was viewed as empowering and person-led and laid the foundations for a transformative model of care and service delivery that moved beyond paternalism [3,13,14]. However, it can be argued that there was a modification, rather than a transformation, in terms of services moving towards this orientation [13,15].

Studies [2,3,16,17,18] highlighted that recovery has an array of meanings. Often, it is determined by the underpinning theoretical knowledge base used by each individual to understand their own experiences of recovery or, in the case of professionals, making sense of each individual’s recovery journey. The different disciplines involved in service delivery view recovery in very different ways, leading to tensions surrounding how people should be supported during their recovery journeys [2,3]. Psychiatry (medication) and psychology (mind/body) often focus on the individual as needing to change [2]. However, social work views the person within their psychosocial circumstances, seeing the whole person through a holistic approach [2,3].

This led to the concept of recovery being viewed as a contested concept [2,3,16,17,18], which is something Pilgrim refers to as a “polyvalent concept” or a “working misunderstanding” [9]. In 2011, a conceptual framework was published for personal recovery based on a systematic review and narrative synthesis [18]. This framework identified five core processes necessary for personal recovery: connectedness, hope and optimism about the future, identity, meaning in life, and empowerment [18]. This was an important contribution because it provided a more specific understanding of what exactly personal recovery is within a mental health context [18].

The concept of personal recovery was first introduced to Irish mental health policy in 2006 [3]. This was evidenced in the national mental health policy at the time, *A Vision for Change: Report of the Expert Group on Mental Health Policy* [3]. At the time, it was highlighted as central to how services should be developed and delivered moving forwards [3]. Key developments since 2006 in Ireland include Advancing Recovery Ireland (ARI) being established in 2012, a national framework for measuring the development of recovery-orientated services being published in 2017 [2], and the current national mental health policy, *Sharing the Vision: A Mental Health Policy for Everyone*, being introduced in 2020. These were important developments for personal recovery becoming a key consideration in current Irish mental health service delivery [19]. In Ireland, there were challenges with the translation of personal recovery from policy to practice in mental health services [2,3].

Furthermore, the profession of social work has been facing significant challenges to its identity and role across practice settings in Ireland and globally [19]. These challenges were heightened during the pandemic, with increasing levels of social and economic inequalities being faced by people receiving and providing social work services [19,20]. The discourse surrounding social work’s identity views the profession as being at a critical juncture, especially in practice settings where neoliberalism, marketisation, and managerialism have continued to increasingly influence and shape service delivery [15,19,20]. In response, there has been a rise in activism from social work since the pandemic, with current debates asking if the profession is converging towards a more critical and radical identity [20].

The lead author of this paper undertook their doctoral research from 2018 to 2022, seeking to explore the concept of recovery within the Irish mental health context. The aim was to discover whether recovery could be better understood by gaining a deeper understanding of service culture. There are no specific studies globally seeking to explore the impact of social constructionism on service culture as it slowly moves towards an orientation of recovery that is focused on a subjective, personalised journey [1,2,21,22,23,24].

The key argument of social constructionism is that individuals’ experiences are the outcomes of their social interactions [25,26,27]. In other words, each person makes sense of their experiences through their engagements, including interactions and relationships. These engagements are made sense of through linguistic exchanges [16,28]. Understanding each subjective experience of personal recovery involves making sense of the language, culture, and historical influences attached to the “discourse” of recovery in mental health service delivery [28]:

*“The central premise of social constructionism is that professional practices are not based on objective or disinterested implementation of scientific practices; rather, they are contextually, and discursively bound constructions made possible by institutional and everyday discourses and practices.”*

*([28], p. 15)*

Therefore, the doctoral study undertaken from 2018 to 2022 explored how different stakeholders construct their subjective reality of what it means to live with a mental illness and recover. It is important to note that the authors of this paper adopt the position of “moderate constructionists”, accepting that there is an independent reality to mental illness that can involve disease [28,29]. Therefore, the aim of this paper seeks to draw on the findings of this research to explore the evidence based on whether social work and personal recovery are converging or diverging paradigms in terms of mental health service delivery in Ireland and internationally. This involved answering the following questions:

1. How do key stakeholders in Irish mental health services construct recovery?
2. What theoretical, philosophical, discursive, and practice perspectives are relevant for implementing a recovery-orientated approach?
3. What are the constraining and enabling factors that influence the implementation of a recovery-orientated approach to practice?

## 2. Materials and Methods

As previously discussed, there are no studies in the mental health literature that specifically focus on exploring recovery in terms of it being a socially constructed experience [2,16,22]. This research was informed by the lead author’s experience of practice, which saw a disconnect between policy on recovery and the reality of how it is being implemented. Furthermore, an earlier qualitative research study completed by the authors in 2017 and published in 2020 [3] focused on exploring, with one multidisciplinary team within Irish adult mental health services, their views on the concept of recovery. The key findings from that study found that all professionals believed that recovery was about a holistic approach, one where service users were provided with support and expertise from all professionals to support their personal recovery journey [3].

However, the reality of this approach to recovery in practice remained absent for many individuals using and providing services within an Irish context. There was a similar experience internationally across mental health systems [4,15,30]. Consequently, this paper focuses on some of the findings from the follow-on doctoral study between 2018 and 2022, which focused on exploring the experiences of key stakeholder groups involved in providing and receiving mental health services in Ireland. The stakeholders involved included policymakers, policy influencers, professionals, service providers, service users, and family members. This article draws on some of the insights from these different groups, especially findings that contribute to answering whether the recovery and social work paradigms are converging or diverging.

The study completed between 2018 and 2022 involved exploring with the different stakeholders their descriptions and understandings of recovery in terms of its translation into practice and everyday life. The research methodology was informed by seeking to understand the meaning attached to individuals’ actions when participating in their everyday social and cultural situations and contexts [31]. Based on this focus, interpretivism was chosen as the most appropriate methodology. Interpretivism views the social world as having meaning for each individual living within it, which is socially created and recreated [32,33]. Therefore, the focus on meaning led to the decision to adopt a qualitative approach to the research design and analysis of the study [31]. Based on the research questions and exploratory focus of the study, a case study design was chosen [34].

The authors adopted a social constructionist lens to make sense of stakeholders’ experiences of recovery. Consequently, there was an acknowledgement that there is no essentialist truth to recovery, irrespective of who was reflecting their experiences (users and providers of mental health services). This informed the decision to use semi-structured interviews as the appropriate method of data collection when seeking to capture the unique individual accounts of each participant [32,35]. Semi-structured interviews were commonly used for previous mental health recovery studies [36,37,38].

Purposive sampling was chosen because it provided a viable way to answer the research study’s question [32,39]. In terms of the recruited sample, two multidisciplinary teams (*n* = 13) participated, with all disciplines involved, including psychiatry, nursing, social work, and occupational therapy. There were 12 service users who were at different stages of their recovery journey involved in the study. In terms of family members and supporters, there was a smaller recruited sample: six in total. Finally, there were a range of policy makers/influencers who participated in the study (*n* = 22). These included people involved in writing, shaping, implementing, and developing policy and services towards a recovery-orientated approach in Ireland.

Thematic analysis was chosen as the most appropriate method of data analysis. It is a flexible type of analysis, especially with large qualitative samples [40]. Furthermore, it facilitated the process of understanding and interpreting the influence of language and social interaction for each participant’s account of recovery [32]. It is also viable to use thematic analysis with a social constructionist position [32,41]. Given the focus on a social constructionist position, including the influence of discourse, discourse analysis was strongly considered. Discourse analysis focuses on power in shaping meaning, something that is also considered within a social constructionist framework [42]. However, the founding researchers of thematic analysis, Braun and Clarke [42], explained that a constructionist epistemology approach to research is compatible with using thematic analysis where the researchers are not solely focusing on discourse. The authors outlined their positionality regarding recovery being a socially constructed experience—there is an acceptance that there is an independent reality beyond discourse. Consequently, this informed the decision to use thematic analysis for this study. Ethical approval was received from the author’s research ethics committee at Trinity College Dublin. Furthermore, approval was received from the relevant health service authorities for the involvement of participants in the study.

## 3. Results

This paper seeks to explore whether the role of social work is converging or diverging towards a personal recovery orientation. This position on recovery is central to mental health policy and service delivery. Some of the key themes that were iteratively identified during the lead author’s doctoral thesis provide insights into where social work can position itself moving forwards. For this paper, the following themes are discussed:

- The reality of personal recovery (Policy)
- Social recovery is a key process to fulfilment (Family/Supporters)
- Entry to and acceptance by society continue to elude so many (Multidisciplinary Teams)
- We also have a place in this world—agency can lead to recovery (Service Users)

Each theme was chosen for a different stakeholder group, which is noted in parentheses. The following subsections discuss the findings in relation to these four outlined themes.

### 3.1. Reality of Personal Recovery (Policy)

Personal recovery should involve individuals experiencing agency and opportunities in their lives. This is facilitated through the necessary support and resources that are available. Services need to provide the pathway for people to experience what their subjective, personalised journey of recovery means to them. There is also a need for people using services to take responsibility for their journey, especially when there is an attainable reality to their recovery. Participants spoke about the importance of their journeys not leading to experiences of insitutionalisation in the community:

*“We need to be really careful that we’re not creating a new institution in the community.”*

*(Policy Influencer Perspective 1)*

*“looking forward and helping people realise their goals and dreams and ambitions… so probably the closure of the institutions is a big deal but it was obviously very important, but we just did the same thing in a smaller place in the community so that had to shift as well.”*

*(Policy Influencer Perspective 2)*

The reality of personal recovery is that it does not place any clear responsibility on society or government to provide the necessary conditions to enable people to attain their subjective realities and wishes related to recovery following illness. This is a frustration for both providers and users of services:

*“whilst the recovery philosophy has permeated the practice of mental health and changed how professionals and patients might see as objectives of engagement, so a better quality of life, the actual ultimate betrayal, the philosophy…dangled in front of their nose on the basis that oh yes you can, you know this is a much better way… if you have schizophrenia and you see your nurse for half an hour every fortnight, the likelihood that you are going to get any recovery in the sense of the broad use of the term, it’s beyond an insult, it’s an injury to that person because you have raised expectations and you have done nothing to actually provide them with the means of, or the infrastructure, that a recovery philosophy would actually give them. So, they don’t have personal assistant, they don’t have you know money, they don’t have places to go to, they don’t have meaningful vocational or occupational… So, while there has been a very useful incorporation of the recovery movement into mental health practice, the worst has happened which is that the philosophy has been trumpeted, but everyone has to suck it up, practitioners who would love to have a much more meaningful and reciprocal and equal personalised engagements with their patients around what it would mean to make their lives better. But instead, we have dwindling resourcing and staffing in mental health services and no prospect, politically or otherwise, in sight of that changing”.*

*(Policy Influencer Perspective 3)*

For providers of services, their experiences of personal recovery often led to feelings of alienation when seeking to translate it from policy to practice. This was also an experience echoed by users of services:

*“There’s also a difficulty in that the word recovery alienates a lot of people who do have a mental health difficulty, who feel that they don’t relate to the idea that they are recovered. So, I’ve certainly had people with self-experience who say to me that they don’t like the word recovery… that they feel that it excludes them if they have on-going mental health challenges. If they live on an on-going basis and struggle with their mental health and have to go to a mental health service regularly, they have to take medication regularly on an on-going basis for most of their lives, they feel that recovery sounds as if they’re all better. Now I understand that’s not what it’s meant… the term doesn’t help with that discussion… that has alienated ironically people who have used mental health services”.*

*(Policy Influencer Perspective 4)*

Not only has alienation been experienced by users and providers of services but the discourse regarding mental health within society has contributed to the further isolation of individuals living with enduring challenges linked to their diagnosis:

*“I think while it’s very good to have an openness about mental health challenges, the reality is you don’t often see a celebrity saying “I’ve got Schizophrenia” or even, you know, Bipolar is slightly more acceptable but, that’s not criticising them for saying about pressures or anxiety…But you don’t hear many celebs or you know influencers or whatever say “Oh yeah I’ve got psychosis.”*

*(Policy Influencer Perspective 5)*

Essentially, people who are viewed as celebrities within our society are not speaking about the enduring reality of mental health challenges, contributing to a sense of recovery as something that is easily attainable.

### 3.2. Social Recovery Is a Key Process to Fulfilment (Family/Supporters)

Family members and supporters spoke about the need for discursive practices that reflect experiences of social recovery:

*“Helping somebody come from a crisis into recovery is by making them feel like a loved member of the community again.”*

*(Key Support Perspective 1)*

Experiences of feeling like a loved member of the community are facilitated through everyday social relationships. The challenges for individuals can often occur when reintegrating into society. There can be a pressure and expectation to participate in everyday normalised interactions:

*“You’ve been surrounded by people, service providers, you know your peers and like that your meals have been provided, the heating was looked after, you didn’t have bills to pay. There’s so many different changes then that you have to look after when you come out of hospital.”*

*(Family Perspective 1)*

Participation in the normalised activities of everyday living is a key part of acceptance in our societies and communities. This can be a challenging experience for people and their families living with mental health challenges. Often, families and their loved ones are not viewed beyond their label of mental illness. Being treated as a normal, human being and seeing the whole person holistically are essential:

*“I just think recovery is… it’s so much broader than just mental health like you know… I think it has a place outside of the Mental Health Services and I would like to see it sitting at a higher level that there would be a stream maybe within all of those areas just to make sure that everything that we deliver from a national and a Government perspective… that we’re looking at the whole person and that we’re making sure that all their needs are being met.”*

*(Family Perspective 1)*

### 3.3. Entry and Acceptance from Society Continues to Elude so Many (Mulitdisciplinary Teams)

Professionals involved in seeking to provide a recovery-oriented service believe there is evidence of a changing culture. However, they are growing frustrated with the inadequate supports available to support the philosophy of personal recovery in practice:

*“What is there out there? We’re very poorly funded as everybody knows. So that in itself has a lot of its own constraints. We ourselves don’t have a Primary Care Centre so that makes it very difficult for us in terms of when we do discharge with the patients we would like to have, you know, following up discharge groups and stuff like that. We don’t have our own place to, you know, we don’t have a purpose-built facility to do everything like that… but that’s down the line in terms of budgeting and funds.”*

*(Community Mental Health Nurse Perspective 1)*

Essentially, there is a disconnect between what policy suggests in terms of recovery and the reality of practice for frontline practitioners. Without the necessary tools and resources, providing service users and families with experiences of real hope and empowerment is often impossible:

*“The idea is great but putting it into practice is another thing as well. So we talk about recovery and this way and that way, but say if somebody has been in hospital, they are discharged home; I work in the community, there’s only so many resources that I have and you try to link them in with community to get them living back to a normal life… we would love to be able to provide a service. Our own local service, as a team and all that. It makes it very difficult. You know in terms of trying to run a group for, trying to do one to one’s and all that sort of stuff. It definitely makes it difficult.”*

*(Community Mental Health Nurse Perspective 1)*

These unrealistic expectations have an impact not only on the experiences of service users and families but also on the professionals seeking to provide the necessary services and supports:

*“The culture’s changing but the actual resources are getting worse… like people don’t have time to do it… well we don’t have the kind of extra resources for people that just can sit down with someone and say “how are you, how’s it going” you know, we don’t. Practically you don’t engage with a patient unless you’ve an agenda. You know you’re here about this, or that or the other. Just say “how are you today”, “lovely day’... treat people as just a normal person. We don’t really have that to be honest you know. And if you were to break the rules and protocols and policies, it would come against you as well in terms of some guy might turn up here and he’s not officially referred and all the rest of it. If you were to ask him to come in and have his dinner because, you know he hasn’t had his dinner, so it’s a different kind of service than it was when I started”*

*(Consultant Psychiatrist 1)*

There have been significant changes to services in recent decades, yet institutional culture and discursive influences have contributed to recovery being out of the reach of most users and service providers. This is further compounded when service users reintegrate into society, and there is no longer the required social support and resources necessary to make personal recovery a reality:

*“I think something like peer support or a nurse, outreach worker that can really dedicate a period of time to practicalities. I think that would make a big difference. Only for a short period of time because it tends to only be for short periods of time. And then there’s long-term pieces with some of the guys that kind of help them along, yeah something like a peer support worker, social care worker, somebody that could take on that role of the day to day. Like I have bills here from people. Sometimes I feel like I’m living their lives, do you know! You’re so involved in people’s lives and like this is basic stuff… That’s all that it needs. So yeah, I think that would be a difference”*

*(Social Work Perspective)*

Recovery is not solely about resources and taking ownership of a recovery plan, it is a much more complex process. For some professionals, the discourse surrounding recovery led to a distorted view of the nature of living with a mental health diagnosis in everyday life:

*“I’ve heard this many times in my clinical practice, people are very unhappy about the concept of recovery because they feel that the mental illness or mental health problems, whichever one you want to call it, psychiatric disorders, they feel that they have been hijacked by a group or cohort of people who are more articulate as you say but also have a lesser or less severe mental health problem”*

*(Consultant Psychiatrist 2)*

### 3.4. We also Have a Place in this World—Agency Can Lead to Recovery (Service Users)

Service users wish to experience fulfilment in their everyday recovery journey. They do not want their everyday lives to be determined by their label or diagnosis of mental illness. Some users of services have found a place in our world, which was facilitated by experiences of agency:

*“Challenges, my freedom was a challenge. It’s just, it’s finding yourself. It’s finding yourself and how you believe that you’ll get there.”*

*(Service User Perspective 1)*

*“It’s a lifelong journey as I said. It’s bumpy. It’s up and down… every day is different… My recovery will be different from your recovery you know. Completely different. It’s just being alert, it’s being… it’s a lifelong battle… takes as long as it needs to be you know”*

*(Service User Perspective 2)*

To be treated as a normal human being and not subordinated by experiences with the everyday oppressive constructs of social reality can support people to have positive experiences of recovery. Service users acknowledge that they have to live with their illness and symptoms but that this should not define their lives when seeking to participate in society. Having a life beyond their diagnosis is possible:

*“Through the assistance of other people and through other perspectives who helped me see my illness in a different framework… I suppose the space and the freedom to really reflect that I actually could achieve just as well as any normal person…diagnosed with a psychotic illness, which I was, I then realised you know actually I can achieve, given the right supports and given the right ways of doing things working with professionals who came from a kind of critical approach to mental illness, or, a holistic approach, not narrow…medical or biomedical model that I was used to it… I’m so lucky that happened because out of that I was able then to achieve. I suppose by also saying that I’m also very aware that you know having a severe vulnerability of course you have to be very aware that is always going to be an underlying problem. But if it’s managed well and if you actually get the right people and the right supports in place, I don’t think you should limit yourself you know, where I initially limited myself too. I’m really glad I didn’t because my whole world was changed and by getting you know higher level degrees and qualifications and I was able to work, and I was able to get more income and I was able to have a better quality of life. All these things helped my recovery, and they helped me, you know my severe mental illness diagnosis wasn’t such a big part of me anymore.”*

*(Service User Perspective 3)*

## 4. Discussion

The experiences of different stakeholders from this study present mixed views of recovery within Irish mental health service delivery. From all perspectives, the challenges faced when making recovery an attainable reality for individuals and their families remain inconsistent. The discursive practices influencing recovery in service culture are continuing to maintain a focus on individualism and personal responsibility. International research further corroborated this reality of recovery [15,30,43,44] with developing evidence that concludes that recovery is becoming a neoliberal entity in mental health systems. Neoliberalism views the economic free market as the best approach to achieving human well-being [45]. This contributed to an experience of individualism for users and providers of services, leading to feelings that they are failing regarding personal recovery. The co-constructed meaning-making that is happening in everyday practice is not leading to any responsibility being taken by services, government, or society. The influence of neoliberalism and individualism also contributed to the need to ask critical questions in terms of societies understanding the difference between mental health challenges and mental illness. Many individuals entering society are often viewed according to their illness or diagnosis in each social encounter [46]. The dominant view in society now is that everyone experiences mental health difficulties [40]. This was further exacerbated during the recent global pandemic [19]. This contributed to people and their families living with mental illness and using services experiencing even further isolation within their communities [20,46].

Interestingly, social work had to respond to similar challenges regarding the influence of neoliberalism [45,47,48]. A recent scoping review [48] looked at the effects of neoliberalism on social work practice in the United State, which was in response to neoliberalism becoming a key discursive influence at all levels of practice. This review [48] highlighted from the outset that the literature is growing regarding the links between social work and neoliberalism. However, there were no studies identified that aimed to focus on the impact of neoliberalism on social work practice. This review also found that research interest has significantly increased in recent years. Yet the need for further studies into the impact of neoliberalism on key social work services, including mental health services, remains [48].

In response to the increasing influence of neoliberalism on social work practice, critical social work approaches have become more prevalent and relevant in responding to issues regarding neoliberalism [47]. Critical social work refers to approaches to practice that seek to overcome both the personal and structural challenges faced by service users [47]. More specifically, Pease [47] outlines the key principles for critical social work practice. Firstly, critical approaches are focused on equality and social justice for marginalised and oppressed individuals and groups in our society. Secondly, there is a focus on working in partnership with these groups in practice. Thirdly, there is a commitment to question the dominant views, interventions, and bodies of knowledge applied in different social contexts. Fourthly, critical approaches consider the position of power in marginalising and oppressing groups. Finally, there is an emphasis on seeking to promote emancipation and social change for individuals and groups in our society.

During COVID-19, an international research study focusing on the role and experiences of social work during the pandemic [20] found that there were increased levels of inequality and marginalisation faced by individuals and groups using social care services. These experiences were shared in relation to increased health inequalities leading to mental health service users’ experiencing more relapses [49]. Essentially, the failings of the free market in achieving well-being for individuals and groups in society (i.e., neoliberalism) [39] was further exemplified and exacerbated during the pandemic. This has important learnings for both social work and recovery in future mental health service delivery.

The findings from this study identified that personal recovery is being influenced and determined by neoliberal discourse. Pease [47] claims that social work needs to adopt an approach to practice that symbolises the following:

*“To foster hope, resilience and dissent in everyday social work practice in the face of consumerist, risk-adverse, marketised, individualising, depoliticising, ideology, policy and social practice.”*

*([47], p. xi)*

If social work can co-construct this meaning-making in everyday mental service delivery, then personal recovery for users and families can begin to lead to positive personal and social change [4,7,19,47,50]. The findings highlighted the need for individuals to have more opportunities for agency when navigating their own recovery journeys. Social work can begin this process by approaching their practice using Fook’s “critical reconstructive process” [50]. Fook developed this four-stage approach in response to the influence of discourse in shaping meaning in our social world [50].

Firstly, there is the process of “desconstruction”, which involves questioning the dominant discourses within a specific social setting or culture. These can be taken-for-granted assumptions about how we interpret the world around us. In the context of this paper, questioning the assumptions regarding personal recovery is the starting point. The findings highlighted that recovery should be a personal journey of fulfilment and moving beyond the label of illness or diagnosis. However, the evidence pointed to a different experience for users and providers of service. The influence of neoliberal discourse contributed to these experiences in practice. According to Fook, “deconstructing involves searching for contradictions, different perspectives and interpretations” ([50], p. 121). Therefore, social work approaching personal recovery from this critical position can support the process of understanding the discourses, power relations, and structures influencing the subjective experiences of service users, families, and service providers [50].

Secondly, by analysing and making sense of the dominant discursive practices and power relations influencing the everyday service interactions and relationships of practice, social work can begin to question these constructions of personal recovery [50]. This stage is known as the “resistance stage”. The types of constructions that should be challenged are those that seek to disempower or hinder agency, such as neoliberal discourses [15,30,43,44]. Thirdly, there is the challenging stage [50]. This involves identifying and labelling the dominant discourses that shape the subjective experiences of personal recovery. Not only is there a focus on challenging the dominant discourses in these experiences but also on which perspectives are missing [50]. For example, it may be suggested that the discourses of paternalism and neoliberalism are shaping the everyday constructions of service delivery. However, missing discourses could include “lived experience”, “co-production”, and a “human rights approach”.

The final stage of Fook’s approach is the reconstruction process. This involves creating new discourses [50]. This process is often informed by identifying the existing, hidden, or missing discourses influencing everyday service delivery regarding recovery. Part of the reconstruction process includes seeking to create service cultures that provide space for new discourses to be created and accepted [50]. This can include expanding the different ways of viewing personal recovery or recovery-orientated services. Fook advises that “formal theories are a form of discourse” that can be introduced to the reconstruction process ([50], p. 126). This can also extend to the introduction of new theoretical approaches that have not been previously used, in this case, personal recovery [50].

One theoretical approach that could be introduced during the reconstruction process regarding personal recovery is social recovery [5,7]. The findings from this study highlighted the limitations of personal recovery, largely due to neoliberal discursive practices [15,30,43,44]. Unfortunately, the reality of current discourse on personal recovery places the responsibility on the individual rather than the collective (i.e., social institutions, state, and society). That is not to say that personal responsibility is not important regarding an individual’s recovery journey [3]. Individuals need to be motivated in relation to their own recovery journey for them to succeed [3].

Social recovery is a relatively new concept and approach to recovery in mental health [4,7]. Social recovery is different from personal recovery because it specifically focuses on how individuals can become active citizens in society, leading to meaning and purpose in their lives [7]. More specifically, it focuses on the collective culture in society, the social relationships that take place, and the interdependent nature of navigating a life not determined by diagnosis or illness [4,7].

Norton and Swords [4], building on the work of Ramon [7], present six key influencers necessary for social recovery to be part of an individual’s recovery journey in the reconstruction process [50]. Firstly, economic stability relates to opportunities for education and employment which can be maintained. This can contribute to an “active” social identity across different social spaces for each person on their recovery journey. The second influence for social recovery is social interactions/connections. The focus here is on facilitating opportunities for engagement in friendships and social groups within an individual’s community [4].

Thirdly, housing is a key parameter for social recovery because it leads to experiences of stability, independence, and safety [4]. These are necessary factors in order for individuals to lead a fulfilling life beyond their mental illness [24]. The fourth influence necessary for social recovery is personal relationships [4]. Often, personal relationships need to be repaired, sustained, and improved when an individual is seeking to move beyond their mental illness and become an active citizen in society. One example can be family relationships. Fifth, social support is identified as key to social recovery. This relates to voluntary support in the community, for example, peer support groups. It can also be supports that are not informed by lived experience but provide opportunities for social relationships that are consistent, responsive, and accepting [4]. Finally, there is purchased support or formal arrangements; examples include service and professional input such as peer support or family support work involvement.

Through the process of reconstruction [50], social recovery discourse can support social workers to challenge the contradictions of the personal recovery culture within mental health services. Some examples of opportunities for social work to approach practice from a social recovery perspective could include psychosocial assessments, multidisciplinary meetings, family meetings, interagency collaborations, and advocacy work. This provides social work with a framework to challenge the current dominant discourses of service delivery, which centre on the individual and the personal, rather than the collective and social [19]. The idea of dissent became a key consideration for critical social work practice [47], with there being clear contradictions seen between the international definition of social work and the reality of practice [51]. Key aims that form part of the global definition of social work include promoting “social change”, “empowerment”, and the “liberation of people” [52]. These outcomes from social work intervention come from collective engagement and intervention alongside people using social work services and seeking to overcome the social structures and inequalities they face within a mental health context regarding their personal recovery journey [2,3,53]. Ultimately, given the skillset of social workers, including a strong commitment to relationship-based practice, advocacy, human rights, and social justice, there is a real opportunity for the profession to converge and transform its recovery-orientated service culture towards a reality that is empowering and fulfilling for all stakeholders.

## 5. Conclusions

Recovery as a personalised journey has become central to mental health service delivery across international jurisdictions. Reflecting on an Irish case study, this paper explored the different ways personal recovery is socially constructed within service culture. This included interviews with a range of stakeholders, including service users, family members, professionals, and policy influencers. The findings illustrated a need for service culture to critically reflect on the reality of recovery for users and providers of services, with neoliberalism being highlighted as a dominant discourse in people’s everyday experiences. Neoliberalism also influenced the profession of social work in recent years across countries, with “critical social work” becoming a key consideration for social work education, practice, and research. Drawing on these developments, combined with the findings from the Irish case study, the authors presented possibilities for the paradigms of social work and personal recovery to converge through the process of reconstruction. Social recovery was explored as a possible new theoretical orientation for transforming the current realities of personal recovery for mental health services. Ultimately, the social work role is best equipped to support this transformation of mental health service culture.

## Data Availability

The data presented in this study are available on request from the corresponding author upon reasonable request. The data are not publicly available due to privacy and ethical issues.
